# Silver Nanoparticles as a Potent Nanopesticide: Toxic Effects and Action Mechanisms on Pest Insects of Agricultural Importance—A Review

**DOI:** 10.3390/molecules29235520

**Published:** 2024-11-22

**Authors:** Daniel Martínez-Cisterna, Olga Rubilar, Gonzalo Tortella, Lingyun Chen, Manuel Chacón-Fuentes, Marcelo Lizama, Pablo Parra, Leonardo Bardehle

**Affiliations:** 1 Doctorado en Ciencias de Recursos Naturales, Facultad de Ciencias Químicas y Recursos Naturales, Universidad de La Frontera, Av. Francisco Salazar 01145, Casilla 54-D, Temuco 4811230, Chile; d.martinez11@ufromail.cl; 2 Centro de Investigación Biotecnológica Aplicada al Medio Ambiente (CIBAMA), Universidad de La Frontera, Av. Francisco Salazar 01145, Casilla 54-D, Temuco 4811230, Chile; gonzalo.tortella@ufrontera.cl; 3 Laboratorio de Química Ecológica, Departamento de Ciencias Químicas y Recursos Naturales, Universidad de La Frontera, Av. Francisco Salazar 01145, Casilla 54-D, Temuco 4811230, Chile; m.lizama04@ufromail.cl; 4 Departamento de Ingeniería Química, Facultad de Ingeniería y Ciencias, Universidad de La Frontera, Av. Francisco Salazar 01145, Casilla 54-D, Temuco 4811230, Chile; 5 Department of Agriculture, Food and Nutritional Sciences, University of Alberta, Edmonton, AB T6G 2P5, Canada; lingyun1@ualberta.ca; 6 Agriaquaculture Nutritional Genomic Center (CGNA), Temuco 4780000, Chile; manuel.chacon@cgna.cl; 7 Doctorado en Ciencias Agroalimentarias, Facultad de Ciencias Agropecuarias y Medioambiente, Universidad de La Frontera, Av. Francisco Salazar 01145, Casilla 54-D, Temuco 4811230, Chile; p.parra03@ufromail.cl; 8 Departamento de Producción Agropecuaria, Facultad de Ciencias Agropecuarias y Medioambiente, Universidad de La Frontera, Av. Francisco Salazar 01145, Casilla 54-D, Temuco 4811230, Chile

**Keywords:** silver nanoparticles, pests, nanopesticide, insects

## Abstract

Nanotechnology has been a promising plant protection discipline in recent years, attributed to the unique physicochemical properties exhibited at the nanoscale. In this context, silver nanoparticles (AgNPs) have been effective in various applications, including medical, industrial, and agronomic, and during the last few years, the control of insect pests has raised great interest. The present review mainly provides updated information about the use of AgNPs elaborated by different synthesis methods, such as biological (plants, microorganisms), physical, and chemical, and their effect against various insect species of agricultural importance belonging to the order Diptera, Coleoptera, Lepidoptera, and Hemiptera. The physiological and toxic effects of applying AgNPs are reported and characterized by developmental problems, mortality, weight reduction, interference with enzymatic activity, and anomalies in the life cycle. Moreover, in the final section, the action mechanisms through which AgNPs act on insects are also discussed, highlighting mechanisms such as alteration of transmembrane permeability, interruption of DNA replication, alteration of protein synthesis, and production of reactive oxygen species (ROS).

## 1. Introduction

Agriculture faces numerous challenges, including climate change, heat stress, drought, and excess humidity, which contribute to the emergence of new pest and pathogen variants, such as insect pests that are responsible for up to 15% of global crop losses. In addition, diseases and weeds lead to a further 40% reduction in worldwide agriculture production [1,2]. With the rapid growth of the global population, the demand for food production has surged, resulting in increased pesticide application. However, indiscriminate agrochemical use has led to ecological risks, food chain contamination, and heightened insect resistance to pesticides [3,4].

Consequently, there is renewed interest in alternative solutions to synthetic pesticides. Nanotechnology holds significant promise in addressing this challenge, offering a path toward modernized agricultural systems. In particular, nanoparticles (NPs) have shown potential in enhancing plant growth and productivity [5,6]. Integrating nanoscale materials into plants enables the controlled, time-regulated release of agrochemicals (e.g., fertilizers, pesticides, and herbicides) and targeted delivery of biomolecules (e.g., nucleotides, proteins, and activators). The most widely recognized applications of nanotechnology in agriculture are nano-fertilizers and nano-pesticides, which aim to boost crop productivity, efficiency, and safety [7,8]. However, despite these advantages, the risks associated with nanomaterials such as potential toxicity to plants, soil, and non-target organisms, along with the possibility of accumulation within the food chain remain unexplored [9,10]. Recent studies emphasize the need for a balanced approach, highlighting that prolonged exposure to nanoparticles, particularly metal-based ones, may lead to adverse environmental impacts and unintended ecological consequences [11,12]. Consequently, advances in nanotechnology across various applications necessitate the development of synthesis methods that are less harmful, more efficient, reliable, and environmentally friendly [13].

Metal nanoparticles have emerged as essential models across numerous scientific and technological domains due to their unique properties, such as a high surface to volume [14]. This property enables the synthesis of smaller nanoparticles (NPs) through various methods, including biological, physical, and chemical processes, allowing precise control over the size, shape, scale, and stability of synthesized nanoparticles [15,16]. The primary advantages of nanoparticles stem from their role as highly effective nanocarriers for bioactive molecules. These nanoparticles can be loaded or encapsulated with conventional agrochemicals or active ingredients, allowing for their slow, controlled, and targeted release over time [17]. In this context, certain nanoparticles have shown potential in controlling arthropods of economic significance, including moths, beetles, lice, ticks, and mosquitoes [18]. Recent studies indicate that the well-established biocidal properties of silver can be significantly enhanced through the application of silver nanoparticles (AgNPs), particularly in arthropod pest management. AgNPs interact with pests through multiple mechanisms, including physical and chemical actions that enable them to penetrate pest exoskeletons, inflict physical damage, and disrupt essential physiological processes [19]. Therefore, drawing on recent literature, this review examines the potential of AgNPs as a novel alternative for controlling various agriculturally important insect pests and explores the mechanisms through which they exert their effects.

## 2. Silver Nanoparticles (AgNPs)

Silver nanoparticles (AgNPs) are colloidal systems with particle sizes ranging from 1 to 1000 nm [20]. These nanoparticles are widely applied across various industries, including agriculture, textiles, and air filtration, due to their high electrical conductivity as well as distinctive magnetic, electrical, and optical properties, manifested by color diversity and easy size modification [21,22,23]. These properties enable their use in biosensors, cryogenic superconductors, catalysts, and antimicrobials. Notably, the high surface area to volume ratio and crystallinity of AgNPs result in more prolonged activity on target organisms compared to bulk materials [24]. Consequently, nano-based biopesticides hold the potential to address the limitations of conventional synthetic pesticides and traditional biopesticides. The stabilization of active ingredients within nanoparticles enables controlled, sustained release, providing effective, long-term pest management with reduced risks associated with synthetic chemicals [25]. Among the various synthesis methods, green synthesis using biological sources offers several advantages over physical and chemical approaches. It is cost effective, avoids the use of toxic chemicals, is straightforward, requires no special conditions, and yields a high product output [26].

Regarding their interaction with pollutants, AgNPs are among the most cost effective for the catalytic reduction of organic pollutants, costing approximately 1/50th of materials like Au and Pt [27]. Conventional pesticides suffer several polluting processes in the soil, such as leaching, causing the active ingredient of the pesticide and its inert ingredients to flow into the groundwater, finding 143 pesticides and 21 of their transformation products [28]. Conventional pesticides also negatively affect non-target organisms such as birds, fish, beneficial insects, and non-target plants [29]. AgNPs exhibit the advantage of forming complexes with protein-rich organic compounds, which contain high concentrations of reducing sugars, polyphenols, and flavonoids that function as reducing agents, creating a stabilizing barrier around the AgNPs surface [30,31,32]. This stabilizing layer, known as a protein corona, reduces free surface energy, thus, decreasing the susceptibility of AgNPs to oxidative corrosion and aggregation in electrolyte solutions [33,34]. The formation of a protein corona typically depends on the synthesis method, such as a biogenic or green synthesis. Biogenic synthesis uses plant extracts as reducing, capping, and stabilizing agents, presenting an environmentally benign alternative to conventional pest management methods that rely on chemical or physical modifications. Moreover, biogenic synthesis is cost-effective, scalable, and minimizes energy consumption and the use of toxic substances [35,36,37]. These methods often involve organisms such as bacteria, fungi, and plants extracts [38].

## 3. Role of AgNPs in Insect Control

**Figure 1:**
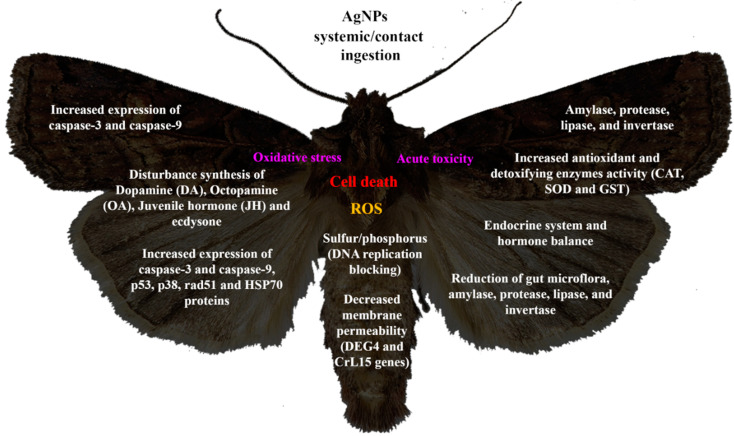
Main toxicity effects of AgNPs against agricultural insect pests. GST: glutatión-S-Transferase; CAT: Catalase; SOD: Super Oxide Dismutase; HSP70: Heat shock protein; CuZnSOD: Copper Super Oxide Dismutase Zinc; DA: Dopamine; OA: Octopamine.

AgNPs have been widely studied for their efficacy against different arthropod pests of economic importance during the last few years. Figure 1 summarizes the reported toxic effects of AgNPs in species belonging to the order Lepidoptera, Diptera, Coleoptera, and Hemiptera.

The efficacy of AgNPs against insects is mainly attributed to their physicochemical properties such as size, surface structure, crystallinity, charge, and catalytic activity, as well as the concentration used. Together, these factors enhance the nanoparticle’s ability to cross biological barriers and induce adverse physiological effects [39]. Inside the cell, AgNPs favor the production of biomarkers of oxidative stress, such as heat shock proteins (Hsp) and endoplasmic reticulum stress, which are directly linked to protein synthesis, causing toxicity and cell death activities such as apoptosis in insects [40]. As shown in Table 1, AgNPs exert a range of effects on various insect orders, detailing the synthesis methods, concentration, particle size, and the mechanisms of action through which they operate.

### 3.1. AgNPs on Lepidoptera

In a study by Manimegalai et al. [41] on *Spodoptera litura* and *Helicoverpa armigera*, AgNPs at a concentration of 150 mg/L exhibited significant antifeedant activity, reaching 78.77% and 82.16%, respectively. The maximum larval mortality rates were 78.49% for *S. litura* and 72.70% for *H. armigera*, with pupal mortality rates of 84.66% and 77.44%. Additionally, the study observed an extended larval development period (13.65 and 14.83 days) and pupal period (15.17 and 17.58 days), alongside morphological abnormalities, including disruptions in epithelial layer (EL) alignment and notable alterations in brush border (BB) and gut lumen (GL) structures. Moreover, a separate study by Yasur and Rani. [42] demonstrated that higher AgNPs concentrations (500–4000 mg/L) led to decreased body weights in the larvae and pupae of *Achaea janata* and *S. litura*. The authors further observed AgNPs accumulation in cellular organelles, which interfered with enzymatic activities, notably CarE (Carboxyl esterases), Glu (Glutathione), GST (Glutathione S-transferases), SOD: Superoxide Dismutase, CAT (Catalase) and POD (peroxidase). These findings suggest that metal ions provoke alterations in detoxification enzymes, such as CAT and POD, which are critical for the removal toxic waste byproducts like H_2_O_2_ and SOD, which acts against anions [43].

Similar results were evidenced in *S. littoralis*, where a concentration of 10 mg/L AgNPs resulted in reduced larval and pupal weight, achieving comparable effects at much lower concentrations than previously tested [44]. Additionally, in a separate study on the same species, AgNPs stabilized with polyvinyl pyrrolidone (PVP) exhibited only an 11.5% mortality rate at the highest tested concentration (600 mg/L), suggesting minimal larvicidal activity for Ag/PVP nanoparticles [45]. In *Heliothis virescens*, AgNPs at a concentration of 100 mg/L delayed pupation and adult emergence, effects linked to AgNP interference with copper transporters essential for insect development [46,47]. For the silkworm *Bombyx mori*, a widely used model arthropod biology, AgNPs were found to increase cocoon growth and weight while inducing intestinal damage [48,49]. Similar intestinal effects have been reported by Jin et al. [50] and Plata-Rueda et al. [51] using diets supplemented with Zinc (Zn) and Chlorpyrifos, respectively. Intestinal damage in arthropods is particularly critical, as the gut is central to metabolism and nutrient absorption. Metal interference within the digestive tract may lead to metal accumulation, preventing entry into the hemolymph and resulting in toxicity [52].

Significant mortality rates have been observed across various species, including *Plutella xylostella* (LC_50_ 0.691 mg/mL), *S. litura* (LC_50_ 0.0312 mg/L–46.9 mg/L), *Earias vittela* (LC_50_ 25.12 mg/L to 45.46 mg/L), *B. mori* (100% mortality at 1600, 3200 mg/L), *Agrotis ipsilon*, and *Trichoplusia ni* (LC_50_ 5.20 mg/mL and LC_50_ 0.81 mg/mL, respectively), with primary toxic effects on larval stages [48,53,54,55,56,57]. These findings indicate that AgNps can induce comparable mortality in Lepidoptera to that of commercial insecticides, such as chlorantraniliprole, flubendiamide, and emamectin benzoate, which exhibit LC_50_ values of 0.56, 3.85, and 6.03 mg/L, respectively, against *S. litura* [58]. For *P. xylostella*, insecticides like spinosad and indoxacarb have demonstrated significant mortality with LC_50_ values of 0.04 and 0.20 mg/L, respectively [59]. Similarly, in *T. ni*, chlorantraniliprole and emamectin benzoate produced LC_50_ values of 0.16 and 0.90 mg/L, revealing potent toxicities comparable to those of AgNPs [60]. These comparisons highlight the potential efficacy of AgNPs as an effective alternative to traditional commercial insecticides for pest management.

### 3.2. AgNPs on Diptera

*Drosophila melanogaster*, a fruit feeding pest during fermentation stages, and *Musca domestica*, a pest found across diverse environments, are both valuable research models due to their suitability for laboratory breeding and their well characterized genomes. AgNP concentrations ranging from 10 mg/L to 200 mg/L have been shown to induce various effects, including interruption of pupal development, reduced larval hatch rates (50 and 80% at 20 mg/L and 40 mg/L, respectively), high mortality rates (97%), decreased larval longevity, and oxidative stress [2,61,62,63]. Comparable developmental disruptions have been documented with commercial pesticides. For instance, in *D. melanogaster*, octopamine receptor (OR) agonists such as Amitraz and Chlordimeform have been associated with delayed development, impaired pupation and adult emergence, reduced egg production, and downregulation of genes linked to female fertility [64]. Additional effects, such as reduced melanin production, fertility impairment, compromised vertical movement capacity, and genotoxicity in *Drosophila* are shown in Table 1. These observations are linked to the toxic action of nanoparticles within the organism, targeting enzymes critical for catabolism [42] and align with findings from other insecticidal studies. Martelli et al. [65] demonstrated that the neonicotinoid insecticide imidacloprid exerts toxic effects on *D. melanogaster*, highlighting oxidative stress as a pivotal factor in insecticide activity and prompting a rapid rise in reactive oxygen species (ROS).

Furthermore, Saini et al. [66] observed indicators of oxidative stress and cell death in *D. melanogaster* following exposure to the nephrotoxins cadmium (Cd) and mercury (Hg), compounds known to disrupt structural integrity and renal function. Similarly, Nas et al. [67] reported that nickel–iron oxide nanocomposites (NiFeO_4_NP) induced genotoxic effects in *D. melanogaster*, evidenced by an increased frequency of wing spots. These findings suggest that AgNPs applied to *Drosophila* can achieve toxicity levels comparable to those of commercial toxic compounds such as Amitraz, chlordimeform, and nephrotoxins like Cd and Hg.

### 3.3. AgNPs on Coleoptera

The effect of AgNPs have also been evaluated on agriculturally important Coleoptera species, such as *Callosobruchus maculatus* and *C. chinensis*, which are common pests of stored legumes. Specifically, AgNPs induced 83% mortality in *C. maculatus*, with LC_50_ values of 2.06 g/kg and 1 g/kg for adults and larvae, respectively. In contrast, the effects were less pronounced in *C. chinensis*, showing a mortality rate of 73% and an LC_50_ of 37.64 mg/L for adults [68]. Ojo et al. [69] reported similar findings with the commercial insecticide NSO (Neem seed oil) on *C. maculatus* larvae, achieving 97% mortality in first-instar larvae, 78.6% in second-instar larvae, 52.8% on third-instar larvae, and 67.4% on fourth-instar larvae. Additionaly, AgNPs caused 20–25% mortality when combined with toluene, and 10–15% when combined with chloroform on *Hylotrupes bajulus*, a pest known to infest conifers such as *Pinus radiata*. Although mortality rates were relatively low, it has been demonstrated that AgNPs functionalized with other compounds can enhance their insecticidal efficacy [70].

Furthermore, the effects of AgNPs synthesized through bioreduction processes involving extracellular metabolites produced by various bacterial isolates, such as *Metarhizium anisopliae*, *Beauveria bassiana*, *Verticillium lecanii*, *Bacillus thuringiensis*, and *B. subtilis*, have been studied on *Rhynchophorus ferrugineus*, a significant coleopteran pest of crops in the Arecaceae family. AgNPs synthesized from *M. anisopliae* demonstrated the highest mortality rates: 90% in eggs, 95% in larvae, and 77% in adults, as reported by Abdel-Raheem et al. [71]. These findings support the efectiveness of biological synthesis methods in producing AgNPs, as noted by Yasur and Rani [42], and highlight their potential for exerting toxic effects across different insect developmental stages.

In studies on *Sitophilus oryzae*, *S. granarius*, and *Trogoderma granarium*, species that infest stored grains, AgNPs achieved 100% mortality in both *Sitophilus* species, while *T. granarium* exhibited mortality rates with LC_50_ values between 4.1 µg/cm^2^ and 11.4 µg/cm^2^ for larvae and adults, respectively. Additionaly, *T. granarium* showed developmental abnormalities, including malformed larvae and pupae, leading to incomplete life cycles [72,73,74]. These observations align with findings by Mantzoukas et al. [75], who demonstrated that entomotoxic proteins, such as leguminous seeds lectins, reduced larval progression to the pupal stage. Collectively, these results suggest that AgNPs can induce high mortality levels in Coleoptera, achieving effectiveness comparable to conventional chemical insecticides.

### 3.4. AgNPs on Hemiptera

In *aphis nerii*, AgNPs exhibited insecticidal effect with an LC_50_ value of 424.67 mg/mL, achieving a maximum mortality rate of 80.55% at the highest concentration tested (700 mg/L) [76]. In *Bemisia tabaci* (whitefly), AgNPs significantly reduced nymph population density, reaching mortality rates of 100%, 100%, 100%, 90% and 80% at intervals of 1, 3-, 7-, 12-, and 21-days post-treatment at a concentration of 3000 mg/L [77]. Similarly, Pushparaj et al. [78] reported high mortality in *B. tabaci* treated with AgNPs at 100 μg/mL, noting pronounced morphological changes in the treated groups. For *Podisus maculiventris*, AgNPs at 181 mg/L slightly extended the time required for pupation and adult emergence, with third-instar nymphs on media containing 100 mg/L AgNPs needing an additional five days to molt into fourth instars. These treatments also resulted in significant reductions in the weights of newly emerged adults, from 55 to 40 mg in males and approximately 75 to 40 mg in females. Additionally, females exposed to AgNPs began oviposition earlier than controls, initiating egg-laying at 12 days of age [46]. In *Lipaphis erysimi*, AgNPs biosynthesized from *B. bassiana* achieved a maximum mortality rate of 60.08% [79].

**Table 1:** Toxicity and mechanism action of AgNps on agricultural insect pests.

Synthesis Method (Size and Concentration)	Especie (Order: Family)	Main Results	Mechanism Action	References
Commercial AgNPs. 209.5 nm. 500, 1000, 2000, and 4000 mg/L	*Achaea janata* (Lepidoptera: Erebidae), *Spodoptera litura* (Lepidoptera: Noctuidae)	Decrease in body weight	CarE, Glu, GST, SOD, CAT, and POD enzyme activity interference	[42]
Green synthesis: Entomopathogenic bacterium *Bacillus thuringiensis kurstaki.* 85 nm. 0.63, 1.25, 2.5, 5, 10, 20 mg/mL	*Agrotis ípsilon* (Lepidoptera: Noctuidae), *Trichoplusia ni* (Lepidoptera: Noctuidae)	LC_50_ of 0.46–0.81 mg/mL for *T. ni*. LC_50_ of 1.95–5.20 mg/mL for *A. ipsilon*	ND	[55]
Commercial AgNPs. 30 nm. 100, 200, 400, 800, 1600, 3200 mg/L	*Bombyx mori* (Lepidoptera: Bombycidae)	Cocoon growth and weight increase (100 and 200 mg/L). Increase in insect growth and mortality (1600 and 3200 mg/L)	Downregulation of expression of Calexcitin-2, cytosolic non-specific dipeptidase, S-formylglutathione hydrolase gene, s-formylglutathione hydrolase gene. Upregulation of Glutathione S-transferase s1 (GSTs1) genes, AK protein, Juvenile hormone binding protein (JHBP), and Isocitrate dehydrogenase.	[48]
Commercial AgNPs. 30 nm. 100, 200 and 400 mg/L.	*Bombyx mori* (Lepidoptera: Bombycidae)	Chaotic crawling, shrinking, disorders in the growth cycle, and diarrhea (100–400 mg/L). Destruction of the basal lamina, expansion of the calciform cells, and deformation of the columnar cells (100–400 mg/L). Reduction in enzyme expression (100 mg/L). Damage at the intestinal level and accumulation of reactive oxygen species (ROS). (400 mg/L)	Upregulation of genes related to location, activity and transmembrane transport (genes DEG-1, DEG-2, DEG-3, DEG-4, DEG-5, and DEG-6). Upregulation of HSP1, Cu/Zn Superoxide Dismutase (SOD), and EF-1. Downregulation of MIOX and ERP57.	[49]
Chemical method with stearic acid. 35–55 nm. 25, 50, 75, 100 and 200 mg/L.	*Earias vittela* (Lepidoptera: Noctuidae)	LC_50_ y LC_90_ of 45.46 y 172.98 mg/L, respectively. Mortality of 93.77% (200 mg/L). LC_50_ and LC_90_ of 25.12 y 160.36 mg/L respectively	Oxidative stress and interference with free thiol groups	[56]
Green synthesis: *Leonotis nepetifolia* leaves extract. 25–53 nm. 30, 60, 90, 120 and 150 mg/L.	*Helicoverpa armigera* (Lepidoptera: Noctuidae), *Spodoptera litura* (Lepidoptera: Noctuidae)	Antifeedant effect of 78.77% and 82.16%. LC50 values at 74.09 mg/L on *S. litura* and 84.58 mg/L on *H. armigera*. Pupicidal activity on *S. litura* (84.66%) and (77.44%) at 150 mg/L. Deformation of ecdysis, pupal malformation, and shrunken pupa. Increase in the larval developmental period (13.65 and 14.83 days, respectively) and pupal duration (15.17 and 17.58 days, respectively)	ND	[41]
Commercial AgNPs. 7–9, 45 nm. 3.5, 35, 100, and 180 mg/L.	*Heliothis virescens* (Lepidoptera: Noctuidae), *Podisus maculiventris* (Hemiptera: Pentatomidae), *Trichoplusia ni* (Lepidoptera: Noctuidae)	It increased adult pupation and hatching time at 180 mg/L on *H. virescens*. Delayed pupation, delayed hatched adults, anormal weigt and sooner oviposition at a concentration of 100 mg/L on *P. maculiventris*.	Increase expression of heat shock protein 70. Increase activities of caspase-3 and caspase-9, markers of apoptosis. Alteration of lipid peroxidation, malondialdehyde, and increased activities of antioxidant enzymes, Glutathione, Superoxide Dismutase, and Catalase. Increase in expression of p53 and p38 proteins. Reduction in tyrosinase activity.	[46]
*Suaeda maritima* aqueous leaf extract. 20–60 nm. 5, 10, 15, 20, 25 mg/L.	*Spodoptera litura* (Lepidoptera: Noctuidae)	LC_50_ of 20.937 (larval instar I) and 46.896 mg/L (pupa). Reduction in egg incubation (100%) to a concentration of 20 mg/L	Neurosecretory cell inhibition, shrinkage of internal cuticle	[57]
Green synthesis: *Punica granatum* peel extract. 14–28 nm. 3.95, 7.8, 15.6, 31.2, 62.5, 125, 250 μg/mL for cellular lines(SF-21) 10, 25, 50, 75 and 100 μg/mL against larvae.	*Spodoptera litura* (Lepidoptera: Noctuidae)	LC_50_ of 31.2 μg/mL. 100% mortality in all larval stages at 100 μg/mL concentration. Reduction in gut microflora (complete inhibition of *Klebsiella pneumoniae*, *Bacillus licheniformis*, *Bacillus cereus*, and *Citrobacter freundi*)	Reduction in the activity of intestinal enzymes such as amylase, protease, lipase, and invertase. Interacting silver ions with the functional groups of nitrogen bases and phosphate groups in DNA and intracellular proteins. Generation of reactive oxygen species, oxidative stress, membrane disruption, protein unfolding, and inflammation.	[54]
Commercial AgNPs. 50–60 nm. 10 mg/mL.	*Spodoptera littoralis* (Lepidoptera: Noctuidae)	Reduction in larval weight gain and pupal weight. Increase in the number of circulating hemocytes	Increase in Glutathione S-Transferase (GST) enzyme activity. Generation of free radicals and ROS, induction of RNA, and DNA damage. Interference with Juvenile hormone (JH) and ecdysone.	[44]
Gamma irradiation with polyvinyl pyrrolidone (PVP). 30 nm. 200 to 600 mg/L.	*Spodoptera littoralis* (Lepidoptera: Noctuidae)	Low toxicity. The maximum mortalities (11.7 and 11.5%) were recorded at 500 and 600 mg/L of Ag/PVP.	Denaturation of proteins containing sulfur or DNA causes the denaturation of insect enzymes. Alteration of gene expression in the midgut, reducing the gut microflora and also amylase, protease, lipase, and invertase activities was reduced and loss of melanin cuticular pigments.	[45]
Green synthesis: *Beauveria bassiana*, *Metarhizium anisopliae*, and *Isaria fumosorosea*. 86.26–257.07 nm. 0.1; 0.3; 0.7; 0.9 and 1.2 mg/mL.	*Plutella xylostella* (Lepidoptera: Plutellidae)	LC_30_ = 0.144 mg/mL, LC_50_ = 0.691 mg/mL, and LC_90_ = 2.011 mg/mL.	ND	[53]
Chemical synthesis (modified Tollens process). 4–24 nm. 10, 20, 40, 60, 80 and 100 mg/L.	*Drosophila melanogaster* (Diptera: Drosophilidae)	LC_100_ at 100 mg/L. Slightly reduction in pigmentation of adult flies (10 mg/L) A 50% decrease in the number of hatched individuals, and all the hatched adult flies had highly reduced body pigmentation (20 mg/L). Reduction in larvae development (60, 80, and 100 mg/L) 97% of larvae were dead, and no pupae were formed (100 mg/L) The long-term exposure to AgNPs influenced the fertility of Drosophila during the first three filial generations	Heat shock stress, generation of free oxygen radicals, and apoptosis. Disturbance of metabolic synthesis pathways of biogenic amines and several reproduction hormones (dopamine (DA), octopamine (OA), juvenile hormone (JH), and ecdysteroids (20-hydroxyecdisone (20HE)). Oocyte maturation delays, degradation of early vitellogenic egg chambers, inhibition of yolk protein gene expression in follicle cells, and accumulation of mature oocytes.	[61]
Commercial AgNPs. 20 nm. 50 µg/mL.	*Drosophila melanogaster* (Diptera: Drosophilidae)	Acute and chronic toxicity	Toxicity in HSP70 protein. Interference with Dopamine (DA), Octopamine (OA).	[80]
Green synthesis: *Olea europaea*, *Ficus carica*, *Eriobotrya japonica*, *Citrus limon*, *Pistacia vera*, *Morus nigra* leaves extract. 5, 8, 10, 18, 22 nm. 10, 50, 100 and 200 mg/L.	*Drosophila melanogaster* (Diptera: Drosophilidae)	Reduction in the number of larvae hatched. Mortality on larvae, pupae, and adults. Reduction in larvae longevity	ND	[2]
Chemical method with sodium citrate. 1–50 nm. 50 mg/L.	*Drosophila melanogaster* (Diptera: Drosophilidae)	Loss of melanin production.	Interruption of the activity of copper-dependent cellular enzymes tyrosinase and copper/zinc Superoxide Dismutase (Cu/ZnSOD)	[81]
Commercial AgNPs. <60 nm. 0.1, 1, 5, 10 mM.	*Drosophila melanogaster* (Diptera: Drosophilidae)	Induction of genotoxic activity	Increased expression of p53 protein and Rad51 affecting critical proteins in the repair of double chain breaks inducing mitochondrial dysfunction and oxidative stress	[62]
Green synthesis: *Manilkara zapota* leaf extract 70–140 nm. 2, 4, 6, 8, 10 mg/mL.	*Musca domestica* (Diptera: Muscidae)	LC_50_ of 3.64 mg/mL and LC_90_ of 7.74 mg/mL. 100% of mortality (10 mg/mL)	ND	[63]
Solvothermal synthesis. 20–60 nm. 1, 1.5, 2, 2.5 g/kg.	*Callosobruchus maculatus* (Coleoptera: Chrysomelidae)	LC_50_ 2.06 for adults. LC_50_ 1.00 for larvae.	Generation of reactive oxygen species and oxidative stress	[68]
Green synthesis: Polyunsaturated fatty acids (linoleic acid), monounsaturated fatty acids (oleic acid), and saturated fatty acids (stearic acid and palmitic acid). 3–13 nm. ND (AgBox).	*Hylotrupes bajulus* (Coleoptera: Cerambycidae)	Agsbox-CL exercised mortality of 20–25%. Agsbox-T exercised mortality of 10–15%	ND	[70]
Green synthesis: *Metarhizium anisopliae*, *Beauveria bassiana*, *Verticillium lecanii* and *Bacillus thuringiensis*, *Bacillus subtilis* culture supernatant. ND. 103, 104, and 105 UFC/mL.	*Rhynchophorus ferrugineus* (Coleoptera: Curculionidae)	90% mortality in eggs, 95% in larvae, and 77% in adults	ND	[71]
Chemical method. 15–31 nm. 25, 50, 75 y 100 kGy	*Sitophilus granarius* (Coleoptera: Curculionidae)	100% mortality for biofilm loaded with 75 kGy of AgNp	ND	[72]
Green synthesis: *Avicennia marina* extract. 15–25 nm. 50, 100, 150, 200 y 250 mg/kg.	*Sitophilus oryzae* (Coleoptera: Curculionidae)	The sum of all the treatments allowed to obtain a mortality of 100% after 4 d	ND	[73]
Green synthesis: *Peganum harmala* L. seeds alkaloids. 22.5–66.2 nm. 3.6, 7.19, 14.37, 28.74, 57.48, 115.0 µg/cm^2^.	*Trogoderma granarium* (Coleoptera: Dermestidae)	LC_50_ between 23.1 and 32.6 µg/cm^2^ against larvae and LC_50_ between 19.6 and 28.4 µg/cm^2^ against the adult. Malformed larvae and pupae.	Disruption of the endocrine system and hormone balance	[47]
Chemical method. ND. 10, 100 y 1000 mg/L.	*Tribolium castaneum* (Coleoptera: Tenebrionidae), *Callosobruchus chinensis* (Coleoptera: Chrysomelidae)	LC_50_ of 15.917 mg/L for *T. castaneum* and LC_50_ of 37.6365 *C. chinensis*. Mortality of 67% for *T. castaneum* and 73% for *C. chinensis*.	ND	[74]
Solvothermal method. 41–46 nm. 300, 371, 458, 566, and 700 mg/mL	*Aphis nerii* (Hemiptera: Aphididae)	LC_50_ of 424.67 mg/mL	ND	[76]
Green synthesis: *Ziziphus* sp. aqueous leaf extract. 44.67 nm. 1000, 2000 and 3000 mg/L	*Bemisia tabaci* (Hemiptera: Aleyrodoidea)	100% mortality at 3000 mg/L	Plasma membrane penetration. Biomolecule breakage. Coagulation of proteins and plasma membrane.	[77]
Green synthesis: *Solanum melongena* leaf extract. 75.14 nm. 2.5, 10, 15, 20, 25, 50 and 100 μg/mL	*Bemisia tabaci* (Hemiptera: Aleyrodoidea)	High mortality at 100 μg/mL	ND	[78]
Green synthesis: *Beauveria bassiana* culture supernatant. 3–25 nm. ND.	*Lipaphis erysimi* (Hemiptera: Aphididae)	Mortality of 90%	ND	[79]

ND: Not determined; LC_50_: medium lethal concentration; LC_90_: lethal concentration 90; HSP70: heat shock protein 70; Agbox-CL: silver boxes with chloroform; Agbox-T: silver boxes with toluene; CarE: Carboxyl esterase enzyme; Glu: Glucosidase; GST: Glutathione S-transferases; SOD: Super Oxide Dismutase; CAT: Catalase; POD: Peroxidase; CFU: Colony Forming Unit; SF-21: *Spodoptera frugiperda*-21 cell line; kGy: Unit (1. 000 Joule/Kg of food.

Based on Table 1, it can be concluded that AgNPs are capable of penetrating arthropod cells, a property attributed to their small size and unique physicochemical characteristics. Furthermore, significant mortality and developmental abnormalities have been observed, indicating that AgNPs are as effective as, or even more efficient than, conventional agrochemicals currently employed in pest control.

## 4. AgNP Action Mechanisms on Insects

**Figure 2:**
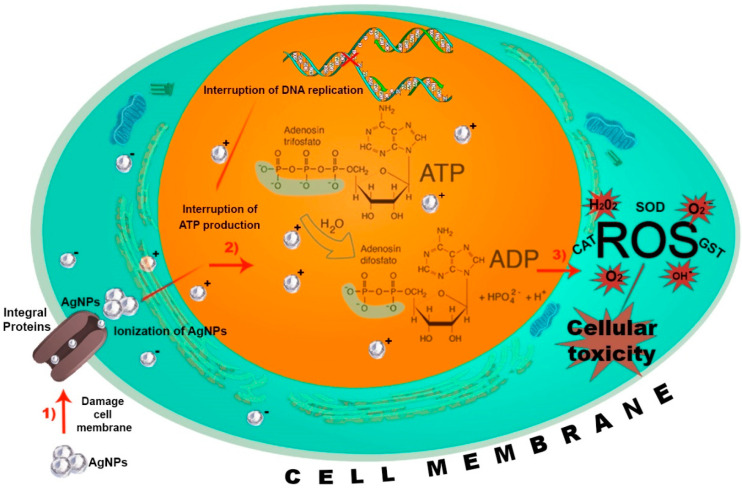
Action mechanisms of AgNPs toxicity. (1) AgNPs cause direct damage to cell membranes, compromising cellular integrity; (2) AgNPs disrupt ATP production, interfering with DNA replication and cellular metabolism; and (3) AgNPs exposure alters protein synthesis and triggers the production of reactive oxygen species (ROS) as a response to cellular toxicity. These mechanisms collectively contribute to the cytotoxic effects of AgNPs. GST: Glutathione S-Transferase; CAT: Catalase; SOD: Super Oxide Dismutase.

The mode of action of any agent within the insect body typically occurs via oral ingestion or direct contact [82]. Current knowledge regarding the mechanisms by which NPs act on insects remains limited. Benelli et al. [83] proposed that nanoparticle toxicity is not solely a function of their physicochemical properties, but rather depends on the number of atoms, ions, or molecules per nanoparticle, which is directly influenced by nanoparticle concentration. This finding is consistent with the effects reported in the main results section of Table 1. Another study suggests that the action mechanisms of NPs vary by classification; for example, the toxicity of nonmetal-based NPs (e.g., SiO_2_ NPs) operates through mechanisms such as desiccation, body wall abrasion, and spiracle blockage, whereas metallic NPs primarily disrupt antioxidant and detoxifying enzyme systems, leading to ROS-mediated apoptosis and DNA damage [84]. Thus, it can be hypothesized that the mechanisms of action differ depending on the type of nanoparticle rather than the target organisms, similar to findings in bacterial studies. Benelli [85] indicated that most research on AgNPs action mechanisms has focused on bacterial models or in vitro cytotoxicity assays. Nosál et al. [86] identified a sequence of mechanisms by which AgNPs exert their effects: AgNPs (1) directly damage cell membranes, (2) uptake free silver ions, leading to disruptions in ATP production and DNA replication, and (3) generate reactive oxygen species by AgNPs in ionic form. These three action mechanisms are illustrated in Figure 2. This section aims to correlate these mechanisms with studies on AgNPs toxicity in insect species, as summarized in Table 1. Additionally, mechanisms observed in other pests will be discussed due to the limited evidence available on agricultural pests.

The first proposed mechanism of action was investigated by Chandrasarekan et al. [87] in *Aedes aegypti* and *Culex quinquefasciatus*, significant insect vectors of diseases such as malaria and zika. In this study, insect membrane permeability decreased following exposure to AgNPs, attributed to the small nanoparticles capable of easily crossing the cell barrier without obstruction, thereby allowing interaction with genes associated with membrane function [88]. Chen et al. [50] identified specific genes involved in this process in *B. mori*. For instance, AgNPs altered the expression of the DEG4 (Slc46a1) gene, which is associated with intracellular transmembrane transport, localization, and transporter activity, ultimately affecting cell membrane functionality.

The second proposed mechanism suggests that AgNPs exert genetic effects on insects by penetrating the exoskeleton and binding to sulfur or phosphorus atoms in proteins, leading to a blockage of translation and transcription processes [87]. These effects were observed in a study by Demir et al. [56] on *D. melanogaster*, where AgNPs increased the expression of p53 and Rad51 proteins essential for the repair of double-stranded DNA breaks resulting in mitochondrial dysfunction, oxidative stress, and subsequent DNA damage. Similar genetic disruptions were reported in the mosquito *Chironomus riparius*, where AgNPs reduced expression of the ribosomal protein gene CrL15, a regulator of ribosomal assembly, thereby impairing protein synthesis [89]. This evidence suggests that AgNPs can induce genetic alterations by interfering with translation and transcription, ultimately impacting protein synthesis. Further research supports these findings in *A. janata* and *S. litura*, where AgNPs caused oxidative stress, leading to DNA damage and increasing the activity of Glutathione S-Transferase (GST), Catalase (CAT), and Superoxide Dismutase (SOD) [42]. GST is an enzyme linked to the appearance of oxidative stress in the body [90]. CAT and SOD are enzymes responsible for maintaining low levels of free radicals to avoid toxicity [89]. Posgai et al. [80] have evidenced that AgNPs are also related to the activity of the protein HSP70 (Heat Shock Protein), which is used as a marker of physiological stress. In this study, *D. melanogaster* exhibited increased expression of HSP70 in response to AgNPs exposure. Additionaly, chronic toxicity was observed across eight generations, evidenced by pigmentation and fertility issues. These effects were primarily linked to disruptions in metabolic systems involving dopamine (DA) and octopamine (OA). This suggests that AgNPs influence DA and OA pathways, which are critical gonadotropin secretion. DA, as the primary precursor for cuticle formation, plays a vital role in insect development, with disruptions leading to developmental issues in subsequent generations [91].

Considering the last background, it is evident that the first two action mechanisms are intrinsically linked to the ROS generation, which subsequently initiates the third mechanism described. Beyond their role in mediating oxidative stress, elevated ROS levels can disrupt melanization (i.e., reduced pigmentation in the insect cuticle). For instance, Armstrong et al. [81] reported that *D. melanogaster* exposed to AgNPs exhibited significantly lighter body pigmentation, with minimal or absent melanin. This phenomenon was attributed to oxidative stress induced by AgNPs, which interfered with copper transporters (Ctr) responsible for copper (Cu) uptake. Consequently, the activity of Cu-dependent enzymes, such as copper/zinc superoxide dismutase (Cu/ZnSOD) and tyrosinase, both essential for melanization, was impaired, resulting in developmental anomalies and disrupted pigmentation [81,92]. These melanization issues suggest that AgNPs may also affect critical hormones and neuropeptides, including juvenile hormone (JH) and ecdysone, in Dipteran insects. These hormones play a pivotal role in insect development and are essential for the regulation of metamorphosis.

Based on the preceding evidence, the present analysis demonstrates the diverse action mechanisms by which AgNPs impact arthropods. It is evident that AgNPs induce toxicity in insects at both cellular and genetic levels, disrupting enzymatic functions and leading to the accumulation of oxidative stress, which adversely affects insect physiology and ultimately results in cell death. However, further investigation into the biochemical mechanisms in insects is necessary to fully elucidate the biological effects of AgNPs on both target and non-target species.

## 5. Conclusions

AgNPs exhibit promising insecticidal properties, offering an alternative to traditional synthetic pesticides widely used in agriculture to combat insect pests. This review identified key impacts of AgNPs on insects’ physiology, including reductions in body weight, pupal malformations, delayed adult emergence, and disrupted melanin production. AgNPs induce these effects primarily by disrupting DNA replication, protein synthesis, and hormonal signaling, and by generating reactive oxygen species (ROS) that interfere with cell membranes and enzyme activity. The unique physicochemical properties of AgNPs may lead to prolonged insecticidal effects, especially through eco-friendly green synthesis methods. However, further studies are essential to assess AgNPs application under field conditions and to better understand their action mechanisms on both target and non-target species. Evaluating the environmental stability, sublethal effects, and potential ecological impact of AgNPs under various biotic and abiotic conditions is also crucial.

Future research should focus on optimizing AgNP formulations to maximize insecticidal efficacy while minimizing risks to non-target organisms and ecosystems. This approach will be essential in developing sustainable, nanoparticle-based pest management solutions for agricultural applications.

## Data Availability

Data are contained within the article.
